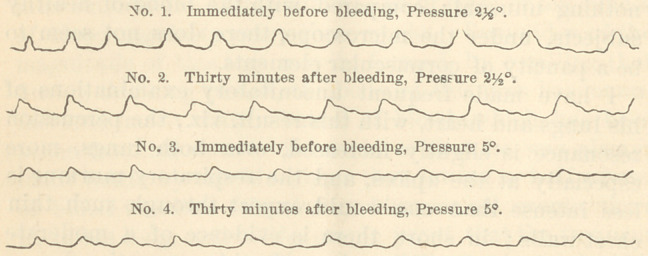# An Instance of Periodical Blood-Losing by Venesection

**Published:** 1875-09

**Authors:** E. Warren Sawyer

**Affiliations:** Chicago


### EXAMINER.

Vol. XXXII. — SEPTEMBER, 1875. — No. 9.

### Original Communications.

### AN INSTANCE OF PERIODICAL BLOOD-LOSING BY VENESECTION.

Read before the Chicago Society of Physicians and Surgeons, Aug. 9, 1875,

By E. WARREN SAWYER, M.D.

Mr. President and Gentlemen :

The case to which I ask your attention for a moment, is an extraordinary instance of habitual venesection, extending through a long period of years.

The subject of this unusual habit is a retired clergyman, now eighty years of age ; and notwithstanding the fact that his blanched face and his slightly stooped shoulders might lead one to conclude that he is a feeble old man, his firm step and keen intellect show an unusual degree of preservation for his advanced years; while his tall stature and immense frame speak of the great physical development which he enjoyed in his youth and early manhood. He has told me that he was an unusually vigorous man, and his entire life has been singularly free from sickness.

He was the son of a farmer, and in his boyhood, worked with his father on the farm. When he was seventeen years old, in the spring of the year, he yielded to the custom which prevailed universally at that period, and was bled for the first time, for no especial reason; this habit of spring time bleeding was followed for the succeeding six years, when he entered college to prepare for the ministry. One of the effects of giving up the active work of a farmer, for the sedentary habits of a student, was a constant heaviness, to relieve which, he resorted oftener to the lancet of his physician, who, in those days, was ever ready to "let blood." During the next ten years, he was bled four to six times each year; always losing from ten to fifteen ounces of venous blood. During the few succeeding years, the frequency of his bleedings was gradually increased, until, at the age of forty, he demanded to be bled once in three weeks; nor has the frequency of the bleedings, or the amount of blood taken, ever grown less. For forty years, has this man suffered the extraordinary loss of eight or ten ounces of blood regularly every three weeks. Some idea of the magnitude of this loss may be formed, by recalling that during this long period, the body of this man has manufactured more than a barrel of blood, which has been taken from his veins! He declares that he was always made better by the bleeding; that letting a half a bowl of blood was like stimulating him; at all events, there is, in his long, active life, ample proof that the frequent blood-letting has never been especially detrimental to his health; for, until he retired from the pulpit, ten years since, he was a hard working minister and for many years a circuit preacher, in Western New York, and frequently was forced to make large circuits on horse-back, exposed to the inclemency of all seasons; still his health has ever been good; nor is he to-day incapable of work —within a month he has assisted in the public dedication of a church in this city.

I made the acquaintance of this gentleman about two and a half years since, in Detroit, when I learned his singular habit; for the past nine months he has been residing with a daughter in this city, and has been under my direction; during this time I have not once failed to bleed him every three weeks, letting from eight to ten ounces of blood.

The demand for the blood-letting is shown by a dyspnœa, which he suffers the last three days and nights of the interlude; so extreme is this, usually, that he is obliged to spend the night in his chair, just before his bleeding day; besides the dyspnœa, there are other evidences of almost venous stasis in the dark, livid lip and the purple finger nails. Immediately after the bleeding, all dyspnœa has disappeared; the lip is red, and the finger nails are no longer purple; besides this, the spirits of the old gentleman seem lighter; he grows talkative; his voice is no longer husky, and he seems in every respect better.

The appearance of the blood which is drawn presents nothing unusual; compared with the blood of healthy subjects, under the microscope, there does not seem to be a paucity of corpuscular elements.

I have made frequent auscultatory examinations of his lungs and heart, with this result, viz., the percussion resonance is slightly increased, over both lungs, more especially at the apices, and the respiratory murmur is less intense than one would expect through such thin chest-walls; in short, there is evidence of a moderate degree of vesicular dilatation. In this connection I may add that he has had, for years, a slight bronchial catarrh, but has never suffered from spasmodic asthma.

I elicited no evidence of organic change in his heart; the sounds are heard feeble but uncomplicated. The action of the heart is not rhythmical; strictly there is no intermission in its revolutions; a contraction of the organ is not omitted, but quite regularly, once in about seven pulsations, there occurs a too short interval between the revolutions; sometimes two of these short intervals are consecutive and then the normal rhythm is observed for a moment. After bleeding, the abnormally short intervals occur less frequently than before.

With a view of learning the effects of the bleeding upon the character of the radial pulse, the effects which were too slight to be appreciated by other modes of examination, I have resorted to the sphygmograph, and have taken tracings, at three different degrees of pressure, both before and after the bleeding.

The tracings taken at 0° pressure, one just before bleeding, the other immediately after the bleeding, are not unlike, and present no distinctive features. But by comparing the tracings taken respectively at pressure 2½° and 5°, one of each taken immediately before bleeding, the others thirty minutes after the bleeding, it is readily seen that the amplitude in the second tracing is greater than in the first; showing that the force of the heart's impulsion, in this instance, is really increased by diminishing the volume of the blood.

No. 1. Immediately before bleeding, Pressure 2½°.

No. 2. Thirty minutes after bleeding, Pressure 2½°.

No. 3. Immediately before bleeding, Pressure 5°.

No. 4. Thirty minutes after bleeding, Pressure 5°.

It is important to observe that the radial artery felt beneath the finger like a hard, round cord, and the artery followed a serpentine course; probably its walls are the seat of atheromatous change.

Further, I may add that the seat of the phlebotomy was always at the bend of the elbow; sometimes the median basilic, at other times the median cephalic vein, of the right or left side, was the vein chosen; and, notwithstanding the cicatrices which marked the innumerable wounds somewhat obscured the anatomical relations of the region, still bleeding was never difficult.

The interesting features which the history of the case contains are, viz.:

*First*, the physiological interest which is attached to the fact that the blood-making function of this man's body has always been unusually active; and further, that there has never been a demand for a peculiar diet, beyond the fact that his appetite for animal food has always been noticeable; his digestion is still unimpaired.

*Second*, the clinical interest which belongs to the fact that the frequent and large losses of blood have never seemed to be hurtful or debilitating.

*Third*, the practical importance of observing great circumspection in repeating venesection at short intervals, else the habit of periodical blood-losing might be established; and this habit, which necessarily subjects the victim to great annoyance, and pain, would, in a less favorable subject, be destructive.

Finally, the question may be asked, could not the habit of periodical blood-losing have been averted in this case? or, when the habit was formed, could it not have been relinquished? The history shows that the first bleeding was not actually demanded; the same is also true of many succeeding bleedings. Undoubtedly there have been periods when it was practicable to break up the habit, either gradually or suddenly; but for many years past, and especially during my acquaintance with the gentleman, I am of the opinion it would have been detrimental to him, and perhaps fatal, to have attempted a reformation: nor is it probable that his future will offer a moment when the habit can be abolished.

## Figures and Tables

**Figure f1:**